# Supplementary figures and images for: A historical legacy of antibiotic utilization on bacterial seed banks in sediments
Source: PeerJ. 2018 Jan 3;6:e4197. doi: 10.7717/peerj.4197 (PMC5756452; doi:10.7717/peerj.4197)

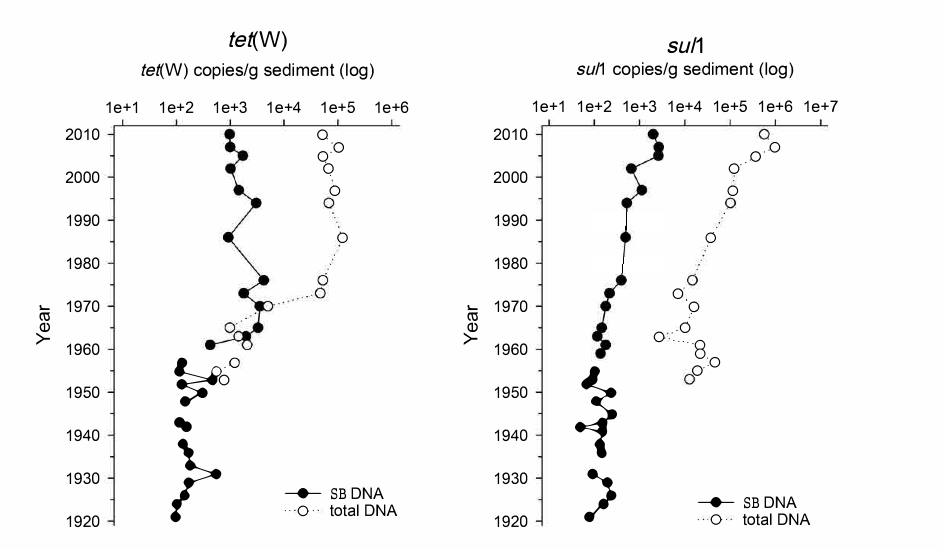

Supplement: Figure S1 — Total abundance (gene copies/g of sediment) of two genes conferring resistance to the antibiotics tetracycline (tet(W)) and sulfonamide (sul1) in sediment samples covering the period between 1920 and 2010 in Lake Geneva, Switzerland. Quantification was made in DNA extracted from the seed bank (SB DNA) and total microbial community (total DNA). [file peerj-06-4197-s001.png]

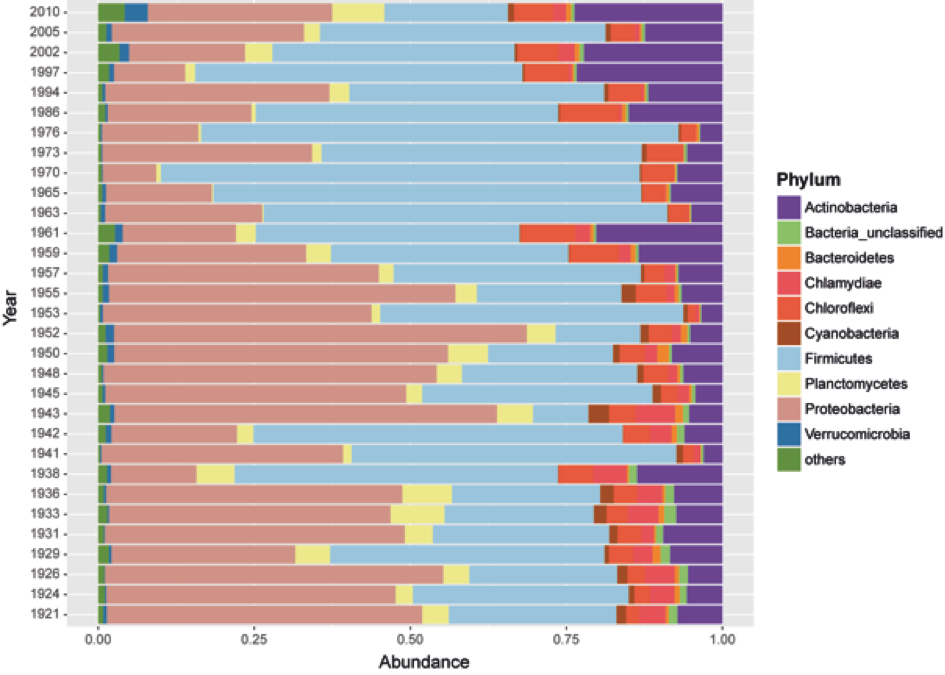

Supplement: Figure S2 [file peerj-06-4197-s002.png]
