# Supplementary material for: A historical legacy of antibiotic utilization on bacterial seed banks in sediments
Source: PeerJ. 2018 Jan 3;6:e4197. doi: 10.7717/peerj.4197 (PMC5756452; doi:10.7717/peerj.4197)
Supplement: Supplemental Information 1 [file peerj-06-4197-s003.pdf]

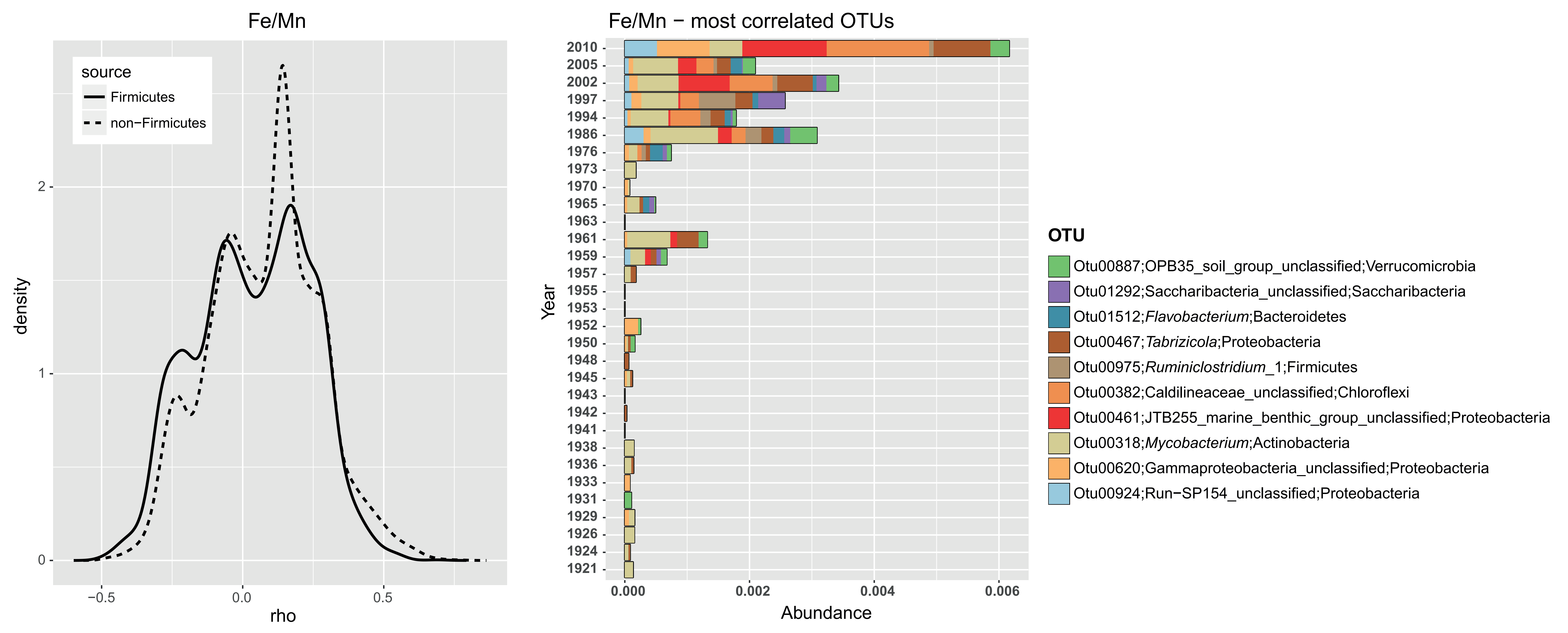
Fe/Mn
source
Firmicutes
non−Firmicutes
density
rho
Fe/Mn − most correlated OTUs
Year
Abundance
OTU
Otu00887;OPB35_soil_group_unclassified;Verrucomicrobia
Otu01292;Saccharibacteria_unclassified;Saccharibacteria
Otu01512;Flavobacterium;Bacteroidetes
Otu00467;Tabrizicola;Proteobacteria
Otu00975;Ruminiclostridium_1;Firmicutes
Otu00382;Caldilineaceae_unclassified;Chloroflexi
Otu00461;JTB255_marine_benthic_group_unclassified;Proteobacteria
Otu00318;Mycobacterium;Actinobacteria
Otu00620;Gammaproteobacteria_unclassified;Proteobacteria
Otu00924;Run−SP154_unclassified;Proteobacteria
