# Supplementary material for: A historical legacy of antibiotic utilization on bacterial seed banks in sediments
Source: PeerJ. 2018 Jan 3;6:e4197. doi: 10.7717/peerj.4197 (PMC5756452; doi:10.7717/peerj.4197)
Supplement: Table S1 — The table is partially adapted from multiple sources (Clatworthy et al 2007, Torok et al 2009, van Hoek et al 2011), including national and international guidelines, as well as personal communication with Swiss and French doctors. [file peerj-06-4197-s004.docx]

Supplementary **Table 1.** Temporal scale showing the respective period when a new antibiotic has been discovered, main period of clinical usage and the approximate year when a first resistance to that compound has been documented. The table is partially adapted from multiple sources(Clatworthy et al 2007, Torok et al 2009, van Hoek et al 2011), including national and international guidelines, as well as personal communication with Swiss and French doctors.

| Antibiotics (class) | Discovery (year) | Period of usage^a^ | Resistance (year)^b^ |
| --- | --- | --- | --- |
| **Sulfonamides** | **1930** | **1940-1960**  **1970-1985**^c^  **2005-2017** | **1940** |
| **Tetracycline** | **1948** | **1955-1970** | **1953** |
| Penicillin | 1929 | 1930-1970  2005-2017^d^ | 1947 |
| Methicillin | 1960 | 1960-2017 | 1962 |
| Ampicillin | 1962 | 1965-2017^e^ | 1974 |
| Cephalosporins | 1960-1970^f^ | 1965-2017^g^ | 1970 |
| Vancomycine | 1957 | 1970-1995  2000-2017^h^ | 1988 |
| Streptomycin (Aminoglycosides) | 1943 | 1946-1960  1980-2000 | 1958 |
| Chloramphenicol | 1947 | 1950-1970 | 1958 |
| Erythromycin (Macrolides) | 1952 | 1995-2010^i^ | 1988 |
| Norfloxacin (Quinolones) | 1979 | 1986-1995^j^ | 1981 |
| Ciprofloxacin (Quinolones) | 1987 | 1990-2005^k^ | 1988 |
| Linezolid | 2000 | 2010-2015 | 2004 |
| Daptomycin | 2004 | 2012-2017 | 2005 |
| Clindamycin (Lincosamides) | 1960 | 1960-1975^l^ | 1964 |

^a^Estimates made for Europe; in some sub-Saharan countries, due to the difficulties of access some antibiotics (such as chloramphenicol and streptomycin) are still largely in use.

^b^Approximate date, mainly adapted from(Clatworthy et al 2007, van Hoek et al 2011). The year of resistance documentation is often much earlier than the year of the description of the mechanism leading to a resistance phenotype. For example for aminoglycosides, the first identified resistance mechanism was the decreased permeability, which was initially described only *in vitro*.

^c^With the availability of cotrimoxazole since 1968, there has been an increased use of cotrimoxazole until about 1985, when 2^nd^ generation quinolones (such as ciprofloxacine) have been largely available.

^d^Recent increase in use of penicillin instead of methicillin or cephalosporins for susceptible strains in order to attempt to reduce selection pressure due to overuse of antibiotics.

^e^Including its use in combination with clavulanate.

^f^Successive discovery of first- second- and third generation of cephalosporins.

^g^There is still a wide use of cephalosporins in Switzerland nowadays, mainly ceftriaxone for the treatment of severe infections due to Gram negative bacteremia, including *E. coli* bacteremia, which represents the most common cause of bacteremia (mainly in the setting of urosepsis).

^h^After a first wide use of vancomycin in initial empirical therapy, especially for severe infections such as endocarditis, bacteremia and fever in neutropenic subjects, the use of vancomycin slightly decreased due to concern about emerging resistance in enterococci; use of vancomycin then again increased due to surge in prevalence of methicillin-resistant *Staphylococcus aureus* (MRSA) and then due to common vancomycin-resistant enterococci (VRE).

^i^Larger usage of macrolides when clarithromycin and azithromycin have been made available, especially for the empirical treatment of lower respiratory tract infections (in combination with cephalosporins, when severe)

^j^Norfloxacin was largely replaced by ciprofloxacin as first line treatment for urinary tract infection from 1995 onwards, due to concern regarding antibiotic resistance and improved efficacy of ciprofloxacin for complicated urinary tract infections; in 2008, the European Medicines Agency recommended to avoid using oral norfloxacin for treatment of urinary infections (http://www.docguide.com/emea-restricts-use-oral-norfloxacin-drugs-utis)

^k^Ciprofloxacin largely replaced norfloxacin for urinary tract infection from 1995 to 2005; in addition from 1990 to 2000, ciprofloxacin was largely used for the empirical treatment of lower respiratory tract infections (LRTI); then due to increased concern about resistance, cotrimoxazole was proposed as first-line empirical antibiotic treatment for uncomplicated urinary tract infection and macrolides replaced quinolones in the treatment of LRTI. This change for a decreasing usage of quinolones was also triggered by the decreased rate of susceptible Gram-negative bacilli to quinolones, which decreased from about 90% in 1990 to about 65-70% in 2000 in USA and in Europe.

^l^Mainly used to treat staphylococcal infections from 1965 to 1975; however, its usage has declined much due to documentation of resistance and due to possible increased risk of post-antibiotic colitis due the broad antimicrobial effect of clindamycin on anaerobes, which constitute more than 90% of the intestinal microbiota.
